# Covid-19 a Strong Predictor of Hyperglycaemia Among Ugandan Patients: A Retrospective Study

**DOI:** 10.21203/rs.3.rs-9012176/v1

**Published:** 2026-05-04

**Authors:** PAUL MUTOO BUKHOTA, MOSES KIRYA, DENIS BWAYO, JOHN PETER MASABA, ASAD BUYINDA, ISMAIL MUTEGEKI, JOHN BOSCO TWINE, PETER OLUPOT-OLUPOT, RICHARD KATURAMU

**Affiliations:** Mbale Regional Referral Hospital; Busitema University; Busitema University; Busitema University; Mbale Regional Referral Hospital; Busitema University; Busitema University; Busitema University; Busitema University

**Keywords:** Prevalence, Clinical characteristics, Hyperglycaemia, Diabetes Mellitus, COVID-19, Eastern Uganda

## Abstract

**Background:**

Hyperglycemia is one of the common complications in COVID-19 patients. Globally, hyperglycemia associated with COVID-19 was estimated to be 25%. Hyperglycemia results in increased morbidity and mortality yet proper screening and management protocols in developing countries. There is paucity of data in developing countries.

**Objectives:**

To determine the prevalence of hyperglycemia, clinical characteristics, and outcomes of COVID-19 admitted patients in Mbale and Soroti regional referral hospitals - Uganda.

**Methodology:**

Retrospective cross-sectional study was conducted on adult admitted COVID-19 patient’s case files at two tertiary hospitals, in Eastern Uganda. 711 COVID-19 patient files with a capillary blood glucose test result during the study period from 1st March 2020 to 31st December 2021 were reviewed. Data was abstracted into a data collection tool specifically designed for this study. The variables included socio-demographics, clinical characteristics, and outcome status of the patients.

Hyperglycaemia was defined based on the COVID-19 management algorithm as capillary blood glucose readings >11.1 mmol/l at or during admission, with the aid of the Glucometer One-Touch©. A primary outcome was hyperglycaemia in hospitalised patients. The Chi-Squared test was used for bivariate analysis, while the logistic regression model was applied for multivariate analysis.

**Results:**

Overall, hyperglycaemia was detected in 459 out of 711 (65%) patients. Living in rural areas (AOR 1.7, 95% CI: 1.1–2.7, P < 0.027), having a medical history of diabetes mellitus (AOR 4.8, 95% CI: 3.4–6.7, P < 0.001), and current use of steroids (AOR 2.9, 95% CI: 1.8–4.7, P < 0.001) immediately before admission were statistically significantly associated with hyperglycemia in COVID-19 patients. COVID-19 found to be an independent risk factor for Hyperglycaemia.

**Conclusion:**

The prevalence of hyperglycaemia among COVID-19 patients in eastern Uganda during the global epidemic was high, at 65%. Pre-admission conditions associated with hyperglycaemia included a medical history of diabetes mellitus, steroid use, living in rural area. Strengthening screening for hyperglycemia and specific management protocols during epidemics and pandemics is recommended

## Background

Coronavirus disease (COVID-19) is an infectious disease caused by the SARS-CoV-2 virus (1–4). COVID-19 was declared a pandemic by the World Health Organization (WHO) on March 11, 2020, in the Republic of China (5). Nearly 509,531,232 million COVID-19 cases had been confirmed globally by August 2023, with 770,437,327 million fatalities (6).

According to the American Association of Diabetes and the American Association of Clinical Endocrinologists consensus, hospital-developed hyperglycaemia is defined as fasting capillary blood glucose ≥ 7.8 mmol/L in a patient with no history or evidence of diabetes, or random capillary blood glucose ≥ 11.1 mmol/L in diabetic patients. (7). Hyperglycaemia was commonly observed among patients admitted with COVID-19 who did not have a prior history of diabetes mellitus and were not using glucocorticoids. (8). Additionally, several studies found COVID-19 infection to be associated with the development of hyperglycaemia or new on-set type 2 diabetes mellitus (DM) (9–11). It has been observed that the prevalence of COVID-19-associated hyperglycaemia with or without pre-existing type 2 diabetes mellitus is 25%, while associated new-onset DM is 19% (12). Data further shows that both critically and non-critically ill COVID-19 patients present with higher than expected blood glucose levels, even in the absence of DM (13).

Among COVID-19 patients, some studies have found hyperglycaemia to be an independent predictor of mortality and morbidity (14). The risk of all causes of death in COVID-19 patients with hyperglycaemia is nearly double compared to that of patients with pre-existing DM (15). Furthermore, hyperglycaemia has been associated with a prolonged hospital stay, often requiring life support with intensive care, mechanical ventilation, and renal replacement therapy (3). Additionally, poor quality of life and non-serious adverse events have also been reported (16). It has been postulated that apart from comorbidities associated with aging, such as hypertension, chronic lung disease, and diabetes, COVID-19 with hyperglycaemia could explain the poor outcomes among elderly COVID-19 patients (17).

There are limited data on COVID-19-associated hyperglycaemia in Sub-Saharan Africa. The only study to assess the prevalence of hyperglycaemia in COVID-19 patients was conducted in South Africa, specifically KwaZulu-Natal, and found that 9.3% of admitted patients had hyperglycaemia (12). This study did not explore the association between different characteristics of COVID-19 patients and hyperglycaemia.

This study aimed to investigate the prevalence of hyperglycaemia, associated factors among admitted COVID-19 patients in eastern Uganda.

## METHODS

This was a retrospective cross sectional study conducted between 1st March 2020 and 31st December 2021.

### Study site

The study was carried out on admitted adult COVID-19 patients at Mbale and Soroti regional referral hospital COVID-19 treatment centres. Mbale and Soroti Regional Referral Hospitals (RRHs) are the highest-tier public referral health facilities located in Eastern Uganda. These two hospitals are among the 13 regional referral hospitals in Uganda. Mbale Regional Referral Hospital (MRRH) is situated at the heart of Mbale City, while Soroti Referral Hospital (SRRH) is a government hospital located in Soroti city in eastern Uganda. More than 90% of the population live in rural communities.

Mbale and Soroti regional referral hospitals were designated by the Ugandan Ministry of Health as COVID-19 treatment centres to admitted and manage COVID-19 patients in March 2020 when COVID-19 was declared a pandemic.

### Study population

The study population included confirmed COVID-19 adult patients admitted in the catchment area of Mbale and Soroti regional referral hospital COVID-19 treatment centres during the period of the study. The Inclusion criteria included all confirmed COVID-19 cases with either positive reverse transcription polymerase chain reaction (RT-PCR) or positive RDT results, COVID-19 patients with documented test results for capillary Blood Sugar at two separate times during the hospital stay and 18 years and above including patients with complete admission files and records while exclusion criteria patients file with a single recording of blood sugar level or no results. All files of COVID-19 patients admitted to these hospitals from 1st March 2020 to 31st December 2021 were retrieved from their archives. A checklist with eligibility criteria was applied to each file.

### Sampling size and Sampling procedure

The medical records with confirmed COVID-19 patients in the archives of Mbale and Soroti Regional Referral Hospital COVID-19 treatment centres were accessed. This was done by sampling of target files with COVID-19 patients above 18 years within the two treatment centres. We used capillary blood glucose (BG), because it is the most commonly used method in clinical practice for blood sugar level monitoring in our setting.

All files of COVID-19 patients admitted to these hospitals from 1st March 2020 to 31st December 2021 were retrieved from their archives. A checklist with eligibility criteria was applied to each file. Files with either, COVID-19 PCR or RDT results and capillary blood glucose measurements on at least two separate occasions were selected for data abstraction. Subsequently, all files were returned to the hospital archives

Infection control procedures such as social distancing, hand washing, and wearing face masks were observed during the data collection period.

### Data collection methods

A data abstraction tool was developed to collect information from patient charts. The tool was adapted from the WHO-modified stepwise questionnaire(4), adopted and contextualised to Ugandan context to collect data on demographics such as age, sex, tribe, education, employment, marital status, residence, weight, laboratory results, biophysical profile, COVID-19 status, and treatment status (18).

Trained research assistants used the data collection tool to collect data. To obtain accurate and complete information from the COVID-19 patients’ files, we trained four research assistants on how to use the selection criteria accurately, record data from the admission forms. The research teams were also trained in infection prevention and control measures for COVID-19, and they used facemasks, eye shields, and hand sanitizers during and after reviewing documents. The principal investigator supervised the data collection exercise from beginning to end, ensuring that every file was checked to confirm the accuracy of entries.

The primary dependable variable was hyperglycaemia. It is a binary either a patient has hyperglycaemia or not recorded as YES for hyper and NO for no hyper

Socio-demographic: Age, weight, height, Education level, Religion, Tribe, Marital status, Occupation, and clinical characteristics: Co-morbidities, type of drugs, clinical features, treatment, symptoms, laboratory results (capillary blood glucose) were the predictor variables.

Trained research assistants extracted data on key variables of interest from confirmed COVID-19 patients’ case files into the study questionnaire. The abstracted data included socio-demographic characteristics, clinical features, laboratory results (Capillary Blood Glucose), treatments administered, and clinical outcomes (death or discharge). The data was then entered into an electronic database using Google Forms.

Completed data tools were reviewed daily by the principal investigator, and all completed tools were stored in a secure location. No names were used as identifiers on the data collection tools. Data capture screens with built-in checks for consistency, logical flow, range, and accuracy of data were designed in Epi-data version 5 and used for electronic data capture (data entry). Data entry was conducted at a secure office at the Busitema University College of Health Sciences, and all data were double-entered. All electronic data was saved on a password-protected external drive. All hard copies of the completed data collection tools were secured under lock and key and were only accessed by key data management team members.

Epi data version 5 was used for data entry. We also used Stata 15 for cleaning and editing our data before data analysis. Once extracted into Excel, the data were sorted to identify exclusion criteria, notably incomplete data (mainly without clinical outcome status). Data processing

The data were cleaned for errors and omissions at all stages of data processing. To ensure data quality, double-entry was performed to ensure correct data entry. Subsequently, the data were exported to Stata Version 15, where sorting, categorization, and necessary variable transformations were conducted.

### Data analysis:

The data were analyzed using Stata (version 15.0, College Station, Texas 77845 USA). Proportions and percentages were determined for categorical variables at the univariate and bivariate levels of analysis. The prevalence of hyperglycaemia in COVID-19 patients was determined and reported as a percentage with 95% confidence intervals. Univariate analysis was used to summarize the clinical and demographic characteristics of our study population. For bivariate analysis, differences between continuous variables were determined using Student’s t-test for normally distributed variables, while differences between categorical variables were determined using the Chi-square test as appropriate.

The 95% confidence interval (CI) was set, with p < 0.05 considered statistically significant. Associations between the dependent variable (hyperglycemia) and independent variables (sociodemographic and clinical characteristics) were initially assessed by bivariate analysis. Multivariate regression models were fitted for all clinical and demographic variables to control for confounding and identify prognostic factors, using death as the outcome variable with a significance level of p = 0.05. In the multivariate analyses, the independent variables were also checked for interaction with a significance level of p < 0.1.

Significant results from the bivariate analysis were used in the multivariate analysis to determine their independent effects on the dependent variables. Results were interpreted using adjusted odds ratios (aORs) and 95% CI. To describe the outcome, a risk analysis was performed using multivariate regression analysis with death as the outcome variable and clinical and demographic characteristics, including hyperglycaemia, as the independent variables.

## RESULTS

Out of 1865 files retrieved from the archive of extracted from both Mbale and Soroti Regional Referral Hospital COVID-19 treatment centres, we extracted retrieved 766 patients’ files and obtain full records for 711 patients’ files were included in this study. The median age of participants was 58 years, interquartile range (IQR) 18 to 98 years. The median age associated with onset of hyperglycemia 57 and interquartile range was 18 to 98.

### Socio-demographic characteristics of COVID-19 patients with hyperglycaemia

As shown in Table 1 there were more males, 53.2% (378/711) and majority 46.1% (328/711) of the patients were aged over 60 years. The majority lived in rural 74.1% (527/711) and were peasants 50.6% (360/711).

Hyperglycaemia increased with age. The proportion of patients with hyperglycaemia was highest in those more than 60 years with 44.2% (203/459)

The proportion of patients with hyperglycaemia was highest in those people who had no education at 34.2% (157/459) and lowest in those who had the highest level of secondary education at 14.2% (65/459).

More peasants were more likely to have hyperglycaemia with a proportion of 47.9% (220/459) while few students presented with hyperglycaemia with a proportion of 4.4% (20/459).

There was also a statistically significant association between hyperglycaemia and place of residence. There was a high proportion of patients from rural areas presenting with hyperglycaemia, 70.4% (323/459) compared to those from urban areas 29.6% (136/459) who were in the rural areas were more likely to present with hyperglycaemia compared to those in Urban area.

### Prevalence of hyperglycaemia among COVID-19 patients

**Figure 1.**
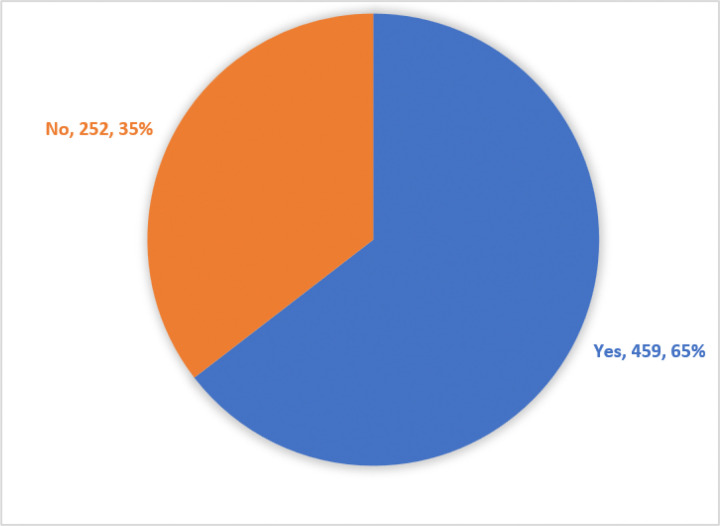
Prevalence of hyperglycaemia among COVID-19 patients

**Figure 2.**
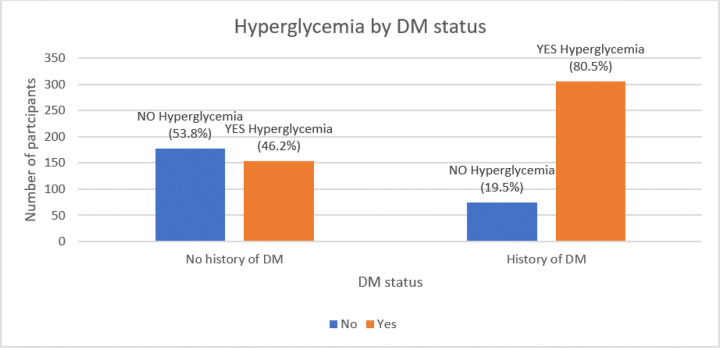
Prevalence of hyperglycaemia by DM status among COVID-19 patients

The overall prevalence of hyperglycaemia among COVID-19 patients was found at (65%: 95%CI 0.6–0.7) (459/771) as shown in Fig. 1 below. However, among those with no history of known DM, the hyperglycaemia prevalence was 46.2% (153/331) while that with known DM it was at 80.5% (306/380) on admission as illustrated in Fig. 2 below.

### Clinical characteristics of COVID-19 patients with hyperglycaemia

Bivariate analysis of clinical characteristics, considering the independent variable and hyperglycaemia using logistic regression, found that having a medical history of diabetes mellitus (COR = 4.8, 95% CI: 3.4–6.7, p < 0.001), hypertension (COR = 2.3, 95% CI: 1.6–3.3, p < 0.001), current use of steroids (COR = 2.9, 95% CI: 1.8–4.7, p < 0.001), and dextrose infusion (COR = 1.8, 95% CI: 1.1–2.9, p = 0.023) before admission were significantly associated with the development of hyperglycemia among COVID-19 patients at a 95% confidence interval (Table 2).

Furthermore, presenting with clinical features such as feeling tired or fatigued or weakness (COR = 2.6, 95% CI: 1.5–4.5, p < 0.001), weight loss (COR = 0.4, 95% CI: 0.3–0.7, p < 0.001), headache or blurred vision (COR = 0.3, 95% CI: 0.2–0.5, p < 0.001), fever (COR = 1.8, 95% CI: 1.1–2.9, p = 0.020), abdominal pain (COR = 8.5, 95% CI: 1.1–64.6, p = 0.014), and coma (COR = 2.8, 95% CI: 1.8–4.4, p < 0.001) were also statistically significantly associated with hyperglycemia among COVID-19 patients.

## DISCUSSION

The study found the prevalence of hyperglycaemia among COVID-19 patients to be 459/711(65%). This is closely similar to other reported studies. A retrospective observational study by viet et al 2022 among 517 COVID-19 adults with hyperglycemia in severe and critical COVID-19 patients in field hospital showed a prevalence of 65.6%(19). This could be attributed to several factors including the study design since we used a retrospective cross sectional study basing on hospital records. Further cross sectional observational study by Ad’hiah et al 2021 among 213 COVID-19 patients reported 22.5% prediabetes and 52.1% diabetes in patients without prior history of diabetes(20) It has been reported by Mohan et al (2021) in Korea among 7341 patients with COVID-19 can lead to glucose dysregulation and induce hyperglycaemia through various mechanisms like inflammation, stress, and direct effects on pancreatic cells (21).

Furthermore, our study also examined the prevalence of hyperglycaemia among COVID-19 patients based on their diabetic status. Among unknown non-DM COVID-19 patients, the prevalence of hyperglycaemia was found to be 46.2%. In contrast, known DM COVID-19 patients had a higher prevalence of hyperglycaemia at 80.5%. These findings align with previous research highlighting that individuals with pre-existing diabetes are more likely to experience hyperglycaemia. A cross sectional study among 7,337 patients with COVID-19 in Hubei Province, China in 2020 by She, Zhu (22) showed that the interaction between COVID-19 and diabetes can worsen glycemic control, leading to increased complications and 7.8% mortality. Another study by Zhu et al. (2020) conducted in China reported similar findings, indicating that hyperglycaemia was more prevalent in diabetic COVID-19 patients compared to non-diabetic patients with an unknown diabetic status. (22, 23).

Additionally, a 46.2% prevalence of hyperglycaemia in previously unknown non-diabetic patients is unexpectedly high. This finding highlights the increased need for monitoring COVID-19 patients, regardless of their medical history of diabetes. The occurrence of hyperglycaemia in non-diabetic COVID-19 patients could be attributed to the reported phenomenon of stress-induced hyperglycaemia, where the body responds to trauma, infections, or sepsis by increasing blood sugar levels through the activation of various neuroendocrine pathways (7).

According to our findings, approximately 53.2% of the patients included in the study were male, indicating a slightly higher representation of males in the study population. This is also similar to a retrospective single centre study by Mazori et al. (2021) conducted among 133 critically ill COVID-19 patients at an Urban academic quaternary care centre, which found that 69% were male patients (24). Another study from Turkey by Tahir Belice et al. revealed that diabetic men had a higher risk of mortality (men 40.9% and women 18.2%), and the rates of admission were higher for men with COVID-19 compared to women (women 17.1% versus men 47.4%) than for other diseases (25).. The reason for this gender distribution could be attributed to various factors, such as differences in exposure to risk factors, or variations in susceptibility to COVID-19 between males and females.

We found that majority, 46.1% of the patients, were over 60 years old. Older age has been consistently identified as a risk factor for severe COVID-19 outcomes in numerous studies worldwide. A retrospective cohort study conducted by Zhou et al. (2020) in Jinyintan and Wuhan pulmonary hospital China on 191 COVID-19 patients reported that advanced age was associated with higher mortality rates 36% among COVID-19 patient (23).

This is also similar to a study by Mazori et al. (2021) conducted among critically ill COVID-19 patients in Malaysia, which found that older age was significantly associated with hyperglycaemia in COVID-19 patients. The prevalence of hyperglycaemia was much higher among older patients 46.6% compared to younger ones (24). Physiological changes associated with aging, such as decreased insulin sensitivity and impaired glucose regulation, contribute to higher glucose levels. Therefore, the higher prevalence of older individuals in the study could reflect the increased vulnerability of this age group to severe COVID-19 disease.

The majority of the patients lived in rural areas 70.4% and were peasants. Similar findings have been reported in other studies. A systematic review by Mehraeen et al. (2020) showed a higher pooled prevalence of 69.8% of hyperglycaemia among COVID-19 patients living in rural areas compared to those living in urban areas. These findings suggest a higher representation of individuals from rural settings and an occupation primarily associated with agricultural work. Rural populations may face unique challenges in terms of access to healthcare facilities, resources, and information. Limited access to quality healthcare in rural areas could potentially impact the detection, management, and outcomes of hyperglycaemia associated with COVID-19. This is in agreement with Mehraeen et al., 2020 studies suggested that limited access to healthcare resources and disparities in healthcare services in rural areas might have contributed to this association (26).

The study identified at the following characteristics; history of DM, hypertension, and cerebrovascular disease. It also reported high prevalence rates of hyperglycaemia in COVID-19 patients with specific chronic illnesses, such as chronic kidney disease. Similar findings were reported by Singh et al. (2020) in a retrospective study conducted in India among 1093 admitted COVID-19 patients in a referral hospital, which found that a history of DM and hypertension were independent risk factors for the development of hyperglycaemia in COVID-19 patients (27).

Additionally, the current use of steroids before admission was also associated with the development of hyperglycaemia. This could be due to the effects of steroids on the body’s glucose metabolism; steroids have been reported to increase the body’s mobilization of blood sugar levels. These findings are consistent with other studies conducted in different populations. Similarly, a systematic review by Jung et al. (2021) in Pakistan, where they conducted aggregate data meta-analyses, trial sequential analyses, network meta-analyses, and individual patient data meta-analyses on 81 clinical trials, identified steroid use as a significant predictor of hyperglycaemia in COVID-19 patients (28). Dextrose contains glucose, so its use directly raises blood sugars. This implies that cautious use of steroids and dextrose in COVID-19 patients is required for good clinical outcomes.

Furthermore, the use of certain other medications, including antiretroviral therapy, antipsychotics, and parenteral nutrition, was associated with a higher prevalence of hyperglycaemia, although the prevalence was relatively lower in patients treated with insulin compared to those treated with oral hypoglycemic agents.

Presenting clinical features such as feeling tired or fatigued, weakness, weight loss, headache or blurred vision, fever, abdominal pain, coma, shortness of breath, and dehydration were significant. Similar findings have been reported in other studies. For instance, a study by Sachdeva et al. (2020) conducted in India found an association between fatigue, weight loss, and hyperglycaemia in COVID-19 patients. The study also reported an increased prevalence of hyperglycaemia in patients with fever and respiratory symptoms (29). Additionally, a retrospective study by Wu et al. (2020) in China found that the elevation of blood glucose levels predicts worse outcomes in hospitalized patients with COVID-19. They identified symptoms such as fatigue, dyspnea, and dehydration as indicators of severe COVID-19 and potential risk factors for hyperglycaemia (30). These clinical symptoms are nonspecific, as they can also occur in other disease conditions. This implies that the detection of hyperglycaemia among COVID-19 patients is not possible solely by clinical symptoms, but rather requires blood sugar level monitoring

The study reported high prevalence rates of hyperglycaemia in COVID-19 patients with specific chronic illnesses, such as cerebrovascular disease, and chronic kidney disease. Furthermore, the use of certain medications, including antiretroviral therapy, antipsychotics, steroids, and parenteral nutrition, was associated with a higher prevalence of hyperglycaemia. However, we could not explain why patients on antipsychotics, and parenteral nutrition were associated with hyperglycaemia. The prevalence was relatively lower in patients treated with insulin compared to those treated with oral hypoglycemic agents. This difference could be explained by the fact that insulin is more potent than oral hypoglycemic agents in controlling blood sugar.

The study found that living in rural areas was associated with a higher risk of developing hyperglycaemia in COVID-19 patients. These findings are consistent with those of Sharma et al. (2021) in the United Kingdom, who demonstrated that urban residency was associated with an increased risk of developing hyperglycaemia among COVID-19 patients (31). One possible explanation for this higher risk could be that urban areas often have different lifestyles and dietary habits compared to rural areas. Urban populations may have a higher consumption of processed foods, junk food, sugary beverages, and sedentary lifestyles, which are known risk factors for hyperglycaemia and diabetes. These unhealthy habits may exacerbate the impact of COVID-19 on glucose regulation and increase the likelihood of hyperglycaemia in infected patients. A study by Mehraeen et al. (2020) conducted in Iran found that living in rural areas was associated with a lower risk of hyperglycaemia in COVID-19 patients (26).

The COVID-19 patients who had a medical history of diabetes mellitus had 4.8 times increased odds of developing hyperglycaemia. The presence of diabetes mellitus can impair glucose regulation, and when coupled with COVID-19 disease, which is a stressor to the body, individuals are more susceptible to hyperglycaemia. These findings align with the existing body of evidence, emphasizing the importance of closely monitoring and managing blood glucose levels in COVID-19 patients with a history of diabetes mellitus. Numerous studies have reported that pre-existing diabetes is a significant risk factor for hyperglycaemia in individuals with COVID-19 (9, 32, 33).

The current use of steroids before admission was associated with 2.9 times higher odds of developing hyperglycaemia in COVID-19 patients. Steroid medications, such as dexamethasone, have been widely used in the treatment of severe COVID-19 cases to reduce inflammation and improve outcomes. However, steroids can also lead to hyperglycaemia by increasing the catabolism of different body metabolites, thereby increasing blood glucose levels. Several studies have reported that steroids, particularly high-dose glucocorticoids, can induce or exacerbate hyperglycaemia by increasing insulin resistance and impairing glucose tolerance (27, 34). These findings have been observed in studies conducted in various populations and settings, including both hospital and community settings. The study also found that COVID-19 patients who were aged 40 years and older and had formal education were associated with a 0.2 lower risk of developing hyperglycaemia. Older age (40 years and older) and formal education may reflect better overall health literacy and a higher likelihood of practicing healthy lifestyle behaviours, which could contribute to better glucose regulation.

### Study strengths

The strengths of this study, it’s the first documented study that assessed hyperglycemia among COVID-19 patients in two public hospitals both urban and rural settings in eastern Uganda thus our results are representative of a bigger population of hospitalized COVID-19 patients in the country.

The sample size was big enough and powered enough to identify potential associations between the variables of interest.

This study looked at capillary blood glucose levels to arrive at the overall prevalence outside ICU settings, compared to several studies that looked at ICU patients.

The research provides potential information in the development of clinical protocol on routine screening for hyperglycemia during epidemics or even pandemics to cater for routine screening and as well as timely assessment and treatment of hyperglycemia and its complications in COVID-19 patients.

### Study limitations

The retrospective nature of the study, obtaining all the data in relation to parameters of interest was not possible. So the results might have been subject to under reporting

There was missing information in some patient’s files which is a challenge especially the patients who were transferred out of the participating hospitals.

## CONCLUSIONS

The prevalence of hyperglycaemia among hospitalised patients was high in our study.

Factors associated with development of hyperglycaemia included increasing age, living in rural areas and male gender while clinical characteristics were history of diabetes mellitus and current use of steroids.

Hyperglycaemia was found to be an independent risk factor among COVID-19 patients.

We therefore recommend that; standardised guidelines for screening and treating hyperglycaemia in all COVID-19 patients and should be incorporated into the routine care package during epidemics and pandemics.

## Figures and Tables

**Table 1:** Socio-demographic characteristics of patients with COVID-19 with hyperglycaemia

Variable	Total n = 711(%)	Hyperglycaemia		COR(95% CI)	P-value
		No n = 252(%)	Yes n = 459(%)		
**Sex**					0.878
Female	333(46.8)	119(47.2)	214(46.6)	1	
Male	378(53.2)	133(52.8)	245(53.4)	1.02(0.8, 1.4)	0.878
**Age (Years)**					0.001
18–29	44(6.2)	6(2.4)	38(8.3)	1	
30–39	69(9.7)	11(4.4)	58(12.6)	0.8(0.3, 2.4)	0.738
40–49	118(16.6)	52(20.6)	66(14.4)	0.2(0.1, 0.5)	0.001
50–59	152(21.4)	58(23.0)	94(20.5)	0.3(0.1, 0.6)	0.004
60+	328(46.1)	125(49.6)	203(44.2)	0.3(0.1, 0.6)	0.003
**Level of education completed**					0.001
No formal schooling	170(23.9)	13(5.2)	157(34.2)	1	
Primary school	277(39.0)	137(54.4)	140(30.5)	0.1(0.04, 0.2)	0.001
Secondary school	116(16.3)	51(20.2)	65(14.2)	0.1(0.1, 0.2)	0.001
Tertiary level	148(20.8)	51(20.2)	97(21.1)	0.2(0.1, 0.3)	0.001
**Employment**					0.001
Business	94(13.2)	34(13.5)	60(13.1)	1.1(0.7,1.9)	0.689
Formal employment	155(21.8)	60(23.8)	95(20.7)	1	
Peasant	360(50.6)	140(55.6)	220(47.9)	1.0(0.7, 1.5)	0.969
Student	21(3.0)	1(0.4)	20(4.4)	12.6(1.7, 96.6)	0.015
Unemployed	81(11.4)	17(6.7)	64(13.9)	2.4(1.3, 4.4)	0.007
**Residence**					0.002
Rural	527(74.1)	204(81.0)	323(70.4)	1	
Urban	184(25.9)	48(19.0)	136(29.6)	1.8(1.2, 2.6)	0.002
**A known DM**	497(69.9)	102(40.5)	395(86.1)	9.1(6.3, 13.1)	0.001

**Table 2:** Clinical characteristics of COVID-19 patients that developed hyperglycaemia

Variable^1^	Total n = 711	Hyperglycaemia		COR(95% CI)	P-value
		No n = 252(%)	Yes n = 459(%)		
**History of**					
Diabetes Mellitus(n = 658)*	380(57.8)	74(29.4)	306(66.7)	4.8(3.4, 6.7)	0.001
Hypertension	216(30.4)	50(19.8)	166(36.2)	2.3(1.6, 3.3)	0.001
chronic heart disease	24(3.4)	5(2.0)	19(4.1)	2.1(0.7, 5.8)	0.137
cerebrovascular disease	13(1.8)	1(0.4)	12(2.6)	6.7(0.9, 52.1)	0.068
chronic lung disease	8(1.1)	2(0.8)	6(1.3)	1.7(0.3, 8.3)	0.539
chronic liver disease(*n = 704*)*	8(1.1)	2(0.8)	6(1.3)	-	0.117
Chronic kidney disease	8(1.1)	1(0.4)	7(1.5)	3.9(0.5, 31.8)	0.205
**Current treatment before Admission**					
Steroids(n = 543) *	132(18.6)	24(9.5)	108(23.5)	2.9(1.8, 4.7)	0.001
Antipsychotics(n = 544) *	8(1.1)	1(0.4)	7(1.5)	3.9(0.5, 31.8)	0.205
Dextrose infusions(n = 538) *	96(13.5)	24(9.5)	72(15.7)	1.8(1.1, 2.9)	0.023
Thiazide diuretics(n = 540) *	11(1.5)	5(2.0)	6(1.3)	0.7(0.2, 2.2)	0.487
Parenteral/Enteral nutrition(n = 545) *	16(2.3)	3(1.2)	13(2.8)	2.4(0.7, 8.6)	0.171
Anti-retroviral therapy(n = 541) *	6(0.8)	0(0.0)	6(1.3)	-	0.068
**Current Treatment for DM during Admission**					
Insulin	292(41.1)	94(37.3)	198(43.1)	1.3(0.9, 1.7)	0.131
Oral anti-hyperglycemic agents	91(12.8)	24(9.5)	67(14.6)	1.6(0.9, 2.7)	0.054
**Symptoms**(n = 704) *					
Frequent urination	23(3.2)	6(2.4)	17(3.7)	1.6(0.6, 4.1)	0.340
Increased thirst(polydipsia)	10(1.4)	4(1.6)	6(1.3)	0.8(0.2, 2.9)	0.762
Increased appetite (polyphagia)	417(58.6)	198(78.6)	219(47.7)	0.2(0.2, 0.4)	0.001
Feeling tired or fatigued or weakness(n = 702) *	Tired	16(6.3)	68(14.8)	2.6(1.5, 4.5)	0.001
weight loss(n = 702) *	512(72.0)	205(81.3)	307(66.9)	0.4(0.3, 0.7)	0.001
Headache or/ Blurred vision(n = 701) *	496(69.8)	210(83.3)	286(62.3)	0.3(0.2, 0.5)	0.001
Fever(n = 705) *	100(14.1)	25(9.9)	75(16.3)	1.8(0.1, 2.9)	0.020
Abdominal pain(n = 699) *	16(2.3)	1(0.4)	15(3.3)	8.5(1.1, 64.6)	0.014
Coma(n = 699) *	142(20.0)	27(10.7)	115(25.1)	2.8(1.8, 4.4)	0.001
Shortness of breath(n = 704) *	26(3.7)	0(0.0)	26(5.7)	-	0.001

* Some data is missing.

1 Multiple response is possible

## Data Availability

The datasets used during our study are available from the corresponding author on reasonable request.

## Abbreviations

ACE2: Angiotensin-converting enzyme 2
CBD: Capillary Blood Glucose
CI: Confidence Interval
COVID-19: SARS-cov-2
CRP: C-reactive protein
CTU: COVID-19 Treatment Unit
DKA: Diabetic Ketoacidosis
DM: Diabetes Mellitus
DNA: deoxyribonucleic acid
FPG: Fasting Plasma Glucose
HDL: High-Density Lipoprotein
HDU: High dependency unit
HHS: Hyperosmolar Hyperglycemic State
ICU: Intensive Care Unit
LDL: Low-Density Lipoprotein
mmHg: millimeter of mercury
mmol/l: millimole per litre
MOH: Ministry of Health
MRRH: Mbale Regional Referral Hospital
OR: Odds Ratio
PCR: Polymerase chain reaction
RBS: Random Blood Sugar
RNA: Ribonucleic Acid
RR: Relative Risk
SD: Standard Deviation
SOPs: Standard Operating Procedures
SRRH: Soroti Regional Referral Hospital
SSA: Sub-Saharan Africa
T2D: Type 2 diabetes mellitus
Tc: Total cholesterol
TG: Triglyceride
UNCST: Uganda National Council of Science and Technology
WHO: World Health Organization
